# Association between skeletal maturation and the developmental stages of the maxillary canine and mandibular second and third molars across different vertical facial patterns

**DOI:** 10.1186/s12903-026-09520-y

**Published:** 2026-08-03

**Authors:** Atefe Rekabi, Mehrnaz Moradinejad, Sepideh Bagheri Hossein Abadi, Maria Mirali

**Affiliations:** 1 Oral and Dental Health Research Center, Ahvaz Jundishapur University of Medical Sciences, Ahvaz, Iran; 2 Department of Orthodontics, School of Dentistry, Ahvaz Jundishapur University of Medical Sciences, Ahvaz, Iran

**Keywords:** Tooth Calcification, Cephalometry, Orthodontics, Biological Markers, Age Factors

## Abstract

**Background:**

Skeletal maturation and dental developmental stages are widely used indicators of biological growth in orthodontics. This study aimed to evaluate the association between skeletal maturation and the developmental stages of the maxillary canine (U3) and mandibular second (L7) and third molars (L8) across different vertical facial patterns (VFPs).

**Methods:**

This cross-sectional observational study assessed lateral cephalometric and panoramic radiographs of patients referred to Ahvaz Jundishapur University of Medical Sciences in 2023–2024. VFP was classified using three cephalometric parameters (LAFH/TAFH, Jarabak index, and FMA) based on a majority rule. Skeletal maturation was determined using the Baccetti cervical vertebral maturation (CVM) method, and dental development was scored according to the modified Gleiser and Hunt system. Data were analyzed using non-parametric tests and PLUM ordinal regression models.

**Results:**

The study sample consisted of 226 participants aged 8–19 years (mean age 12.95 ± 2.39 years), including 155 females and 71 males. Skeletal maturation showed significant positive correlations with U3, L7, and L8 developmental stages across all facial patterns (*p* < 0.001), with L7 showing the highest coefficients. In fully adjusted ordinal regression models, chronological age (CA) was the strongest predictor of U3, L7, and L8 developmental stages (*p* < 0.001). Skeletal maturity remained independently associated only with L7 development (*p* = 0.004). VFP showed no independent significant association with dental developmental stages (*p* > 0.05).

**Conclusion:**

Skeletal maturation was significantly associated with dental development of U3, L7, and L8, with L7 showing the strongest correspondence. In fully adjusted models, dental maturation showed no independent significant association with VFP, while CA remained the main determinant of dental developmental stage.

## Background

In contemporary orthodontics, particularly over the past decade, there has been an increasing emphasis on precise growth assessment to optimize the timing and effectiveness of treatment interventions [1, 2]. The concurrent assessment of skeletal maturation and dental developmental stages, as two pivotal indicators of craniofacial growth, occupies a central role in this context. Skeletal maturation, most commonly evaluated through cervical vertebral maturation (CVM), is regarded as a standard tool for identifying the adolescent growth spurt and timing complex orthodontic interventions such as facemask therapy, headgear, and orthognathic surgery [3]. In parallel, the developmental stages of permanent teeth, particularly the maxillary canine and the mandibular second and third molars, not only reflect an individual’s biological age but also serve as valuable adjunctive markers for assessing maxillofacial growth patterns and planning treatment timing [4, 5].

Dental developmental stages have been operationalized through standardized methods such as the Demirjian system, which scores crown and root formation of seven permanent teeth, and Nolla’s method, which classifies tooth calcification into ten discrete stages. These approaches provide relatively stable biological indicators for estimating maturational age [6]. A growing body of evidence indicates that the calcification stages of the maxillary canine (U3) exhibit a significant association with CVM stages and may be used as a supplementary index to inform the optimal timing of functional or growth-modifying therapies [7]. In addition, the calcification status of the mandibular second and third molars has been reported to yield valuable information in the late phases of skeletal and dentoalveolar maturation, when the craniofacial system approaches its final developmental stages [5].

Studies conducted across different populations have consistently demonstrated a meaningful correlation between dental calcification stages and skeletal maturation indices. For instance, investigations in a Saudi population have shown that the developmental stages of the maxillary canine and third molar can predict CVM phases with acceptable accuracy [8]. Likewise, panoramic radiographic assessments that integrate multiple developing teeth have been proposed as a relatively non-invasive, low-radiation approach for approximating the timing of the pubertal growth spurt, potentially reducing the need for additional radiographic exposures in routine clinical practice [9].

VFPs, typically categorized as hyperdivergent (long face), normodivergent (normal face), and hypodivergent (short face), represent another key variable in craniofacial analysis and may influence both the timing and trajectory of skeletal and dental development [10]. In a recent systematic review, de Souza et al. reported that individuals with a predominantly vertical growth pattern may exhibit altered dental maturation timing compared with those displaying normal or horizontal patterns, an observation that, if validated, could have important implications for the timing of orthodontic interventions. They also highlighted the presence of ethnic and geographic variability in skeletal and dental developmental patterns, underscoring the need for population-specific reference data [11]. Consistent with this notion, evidence from an Iranian cohort showed a strong correlation between dental age (Nolla’s method) and CVM stages, supporting the applicability of these indices in Iranian children, although limitations in sample size and ethnic diversity restrict the generalizability of those findings [12].

Taken together, current evidence suggests that the combined use of multiple indicators, namely, chronological age (CA), dental age, and CVM, provides greater accuracy in identifying the peak growth phase than reliance on any single parameter alone [13]. In recent years, multivariable statistical models and integrative approaches have been proposed to synthesize skeletal and dental information, thereby enhancing the precision of growth assessment and improving guidance for the timing of functional treatments such as facemask and headgear therapy [14]. Nevertheless, there is a relative paucity of studies that simultaneously examine the relationship between skeletal maturation and the developmental stages of the maxillary canine and mandibular second and third molars within the framework of distinct VFPs, particularly in Iranian populations.

Against this background, the present study was designed to investigate the association between skeletal maturation stages and the developmental stages of the maxillary canine and the mandibular second and third molars across different VFPs (hyperdivergent, normodivergent, and hypodivergent).

## Methods

### Sample Size and Patient Recruitment

This cross-sectional observational study was conducted on an Iranian population aged 8 to 19 years who visited the Department of Orthodontics at Jundishapur University of Medical Sciences between 2023 and 2024. All eligible participants were selected based on predefined criteria, and only those with complete clinical records and high-quality digital panoramic and lateral cephalometric radiographs obtained on the same day were included.

The final sample size was determined using Fisher’s z transformation formula for correlation analysis (Eq. 1) [15]. The calculation was based on a conservative significance level of α = 0.01, statistical power of 95%, Z-values of Z_1−α/2_ = 2.57 and Z_1−β_ = 1.65, and an expected correlation coefficient of *r* = 0.70, yielding approximately 27 participants, conservatively rounded up to 28 participants per category. After accounting for a 20% potential attrition or incomplete records, the required sample size was increased to 34 participants per category, corresponding to 68 participants for the two sex groups. Considering three VFP categories, the minimum required sample size was calculated as 204 participants. However, because the study utilized a convenience sampling approach, all eligible individuals who met the inclusion criteria during the recruitment period were included, resulting in a final sample of 226 participants (Female = 155 and Male = 71).

1 $$\:n=\frac{{({z}_{1-\frac{\alpha\:}{2}}+{z}_{1-\beta\:})}^{2}}{{\left(\frac{1}{2}Ln\frac{1+r}{1-r}\right)}^{2}}+3$$

### Inclusion and exclusion criteria

A set of predefined inclusion and exclusion criteria was applied to ensure the selection of individuals with appropriate clinical and radiographic characteristics.

Inclusion criteria:

- Iranian patients between 8 and 19 years of age.
- High-quality digital panoramic and lateral cephalometric radiographs captured on the same day.
- Absence of extracted teeth or dental anomalies.
- No history of orthodontic treatment, trauma, or surgery involving the face or neck.
- No craniofacial anomalies such as cleft lip or palate.
- No systemic diseases affecting growth or development (e.g., endocrine disorders).
- No cervical vertebral anomalies.

Exclusion criteria:

- Poor-quality radiographic images.
- Incomplete patient records.
- Presence of impacted teeth.
- Presence of missing teeth.
- Any radiographic evidence of orthodontic appliances or residual orthodontic hardware.

### Radiographic data collection and CA assessment

Panoramic and lateral cephalometric radiographs were retrieved from the radiology archive of the School of Dentistry, Ahvaz Jundishapur University of Medical Sciences. The radiographs were obtained using a Carestream CS800 unit (United States). Radiographic assessments were performed manually. All assessments were performed by two orthodontic specialists with 4 and 14 years of clinical experience, respectively. Before the main evaluation, the examiners were calibrated using a standardized assessment protocol. Landmark identification, cephalometric measurements, CVM staging, and dental developmental staging criteria were reviewed, and disagreements were discussed and resolved by consensus. For manual cephalometric tracing, radiographs were viewed on a 17-inch Samsung SyncMaster 793DFX monitor (South Korea), and tracing paper was used to identify landmarks and construct the required linear and angular measurements.

**Fig. 1:**
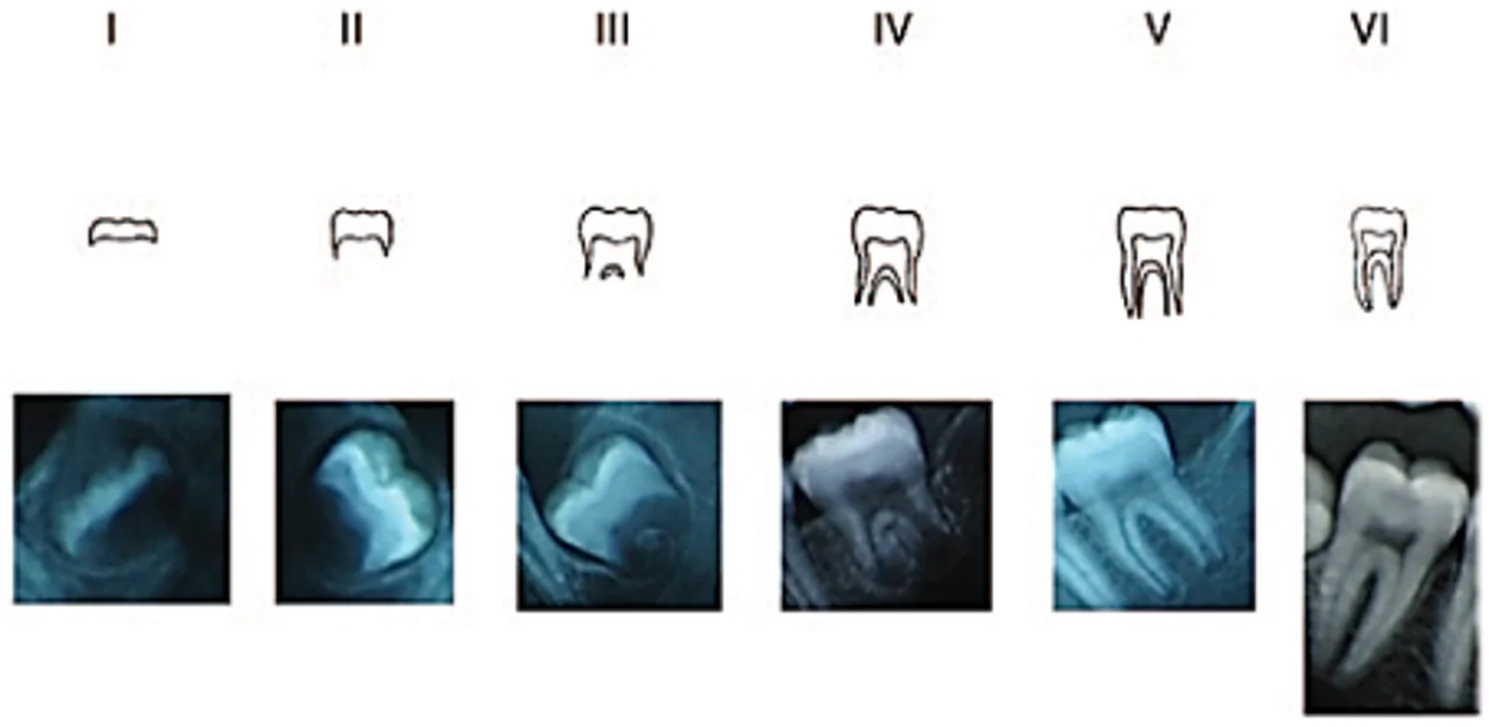
Illustration of the calcification stages of molars [16]

**Fig. 2:**
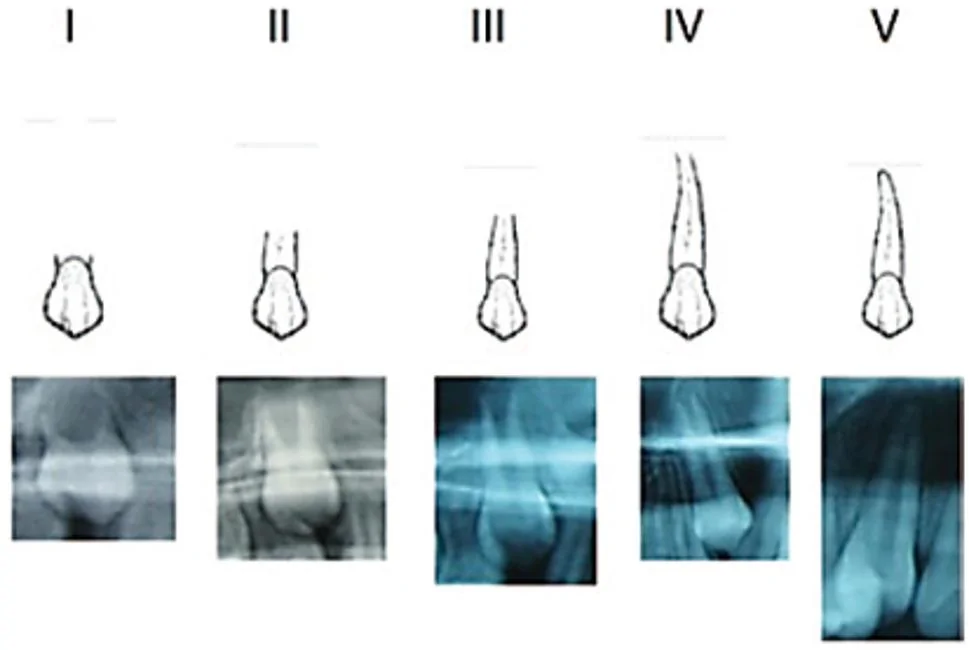
Illustration of the calcification stages for the canine [16]

**Fig. 3:**
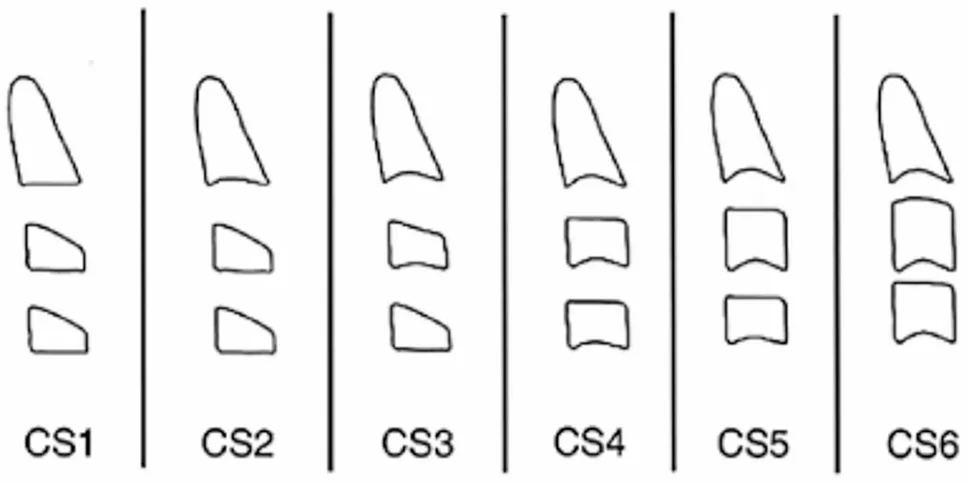
Illustration of the morphologic characteristics of vertebrae C2 through C4 [17]

Accordingly, dental maturation was evaluated based on the calcification stages of the maxillary right canine and the mandibular left second and third molars on the panoramic radiographs, following the modified Gleiser and Hunt method [16] (Fig. 1), which categorizes the canine into five developmental stages and the molars into six (Fig. 2). Building upon the radiographic assessment of dental development, skeletal maturation was subsequently evaluated using the Cervical Vertebral Maturation Staging (CVMS) protocol proposed by Baccetti et al. [17], wherein the morphological characteristics of the C2, C3, and C4 vertebrae are systematically classified into six stages (CVMS I–VI) (Fig. 3). To complement these developmental indicators, CA was calculated by subtracting each patient’s date of birth from the date on which the radiographs were obtained, thereby providing an additional temporal reference for growth evaluation.

### VFP classification

In this study, three cephalometric indicators were utilized to classify the VFP, providing a comprehensive assessment of facial height proportions and craniofacial growth direction. These indicators included the Lower Anterior Facial Height to Total Anterior Facial Height (LAFH/TAFH) ratio, the Jarabak Index, and the Frankfort–Mandibular Plane Angle (FMA). To establish the final vertical facial classification, the results of these three parameters were integrated; when at least two indicators corresponded to the same category, that category was designated as the predominant vertical pattern. This combined diagnostic strategy improved accuracy by minimizing the limitations and potential bias associated with relying on a single cephalometric measurement. The three indicators are described in detail below [10]. In cases where all three indices indicated different vertical facial pattern categories, the measurements were reassessed to exclude tracing or measurement errors. If the discrepancy persisted and no majority classification could be established, the final vertical facial pattern was determined based on the LAFH/TAFH ratio, representing the proportional contribution of the lower anterior facial height to the total anterior facial height.

### LAFH/TAFH ratio

**Fig. 4:**
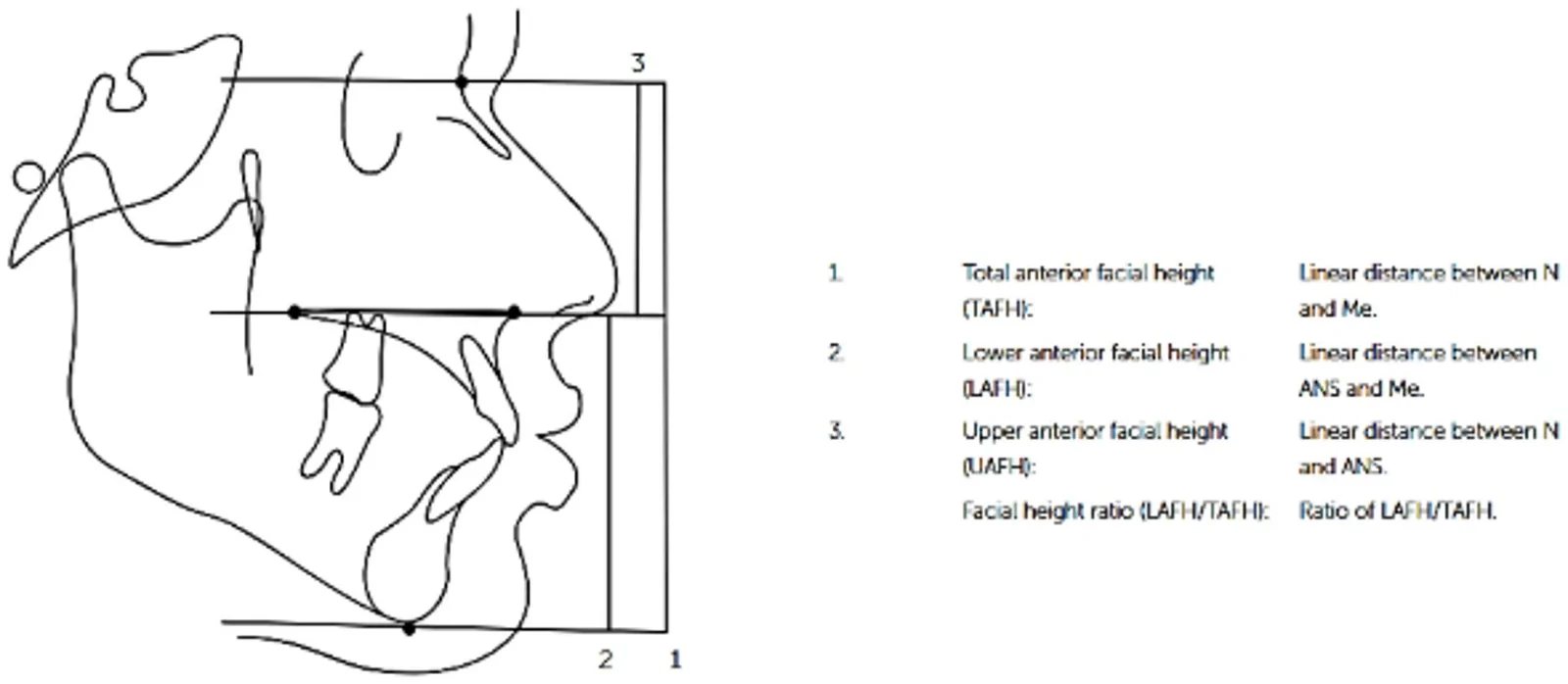
Linear skeletal measurements [10]

For the classification of VFPs, the LAFH/TAFH was used as the primary cephalometric indicator. The relevant anatomical landmarks were identified on the lateral cephalometric radiographs, and the corresponding linear measurements were obtained. Based on the calculated ratio, individuals were categorized into three groups: a normodivergent pattern (50–55%), a hypodivergent pattern (< 50%), and a hyperdivergent pattern (> 55%) (Fig. 4) [10].

### Jarabak index

**Fig. 5:**
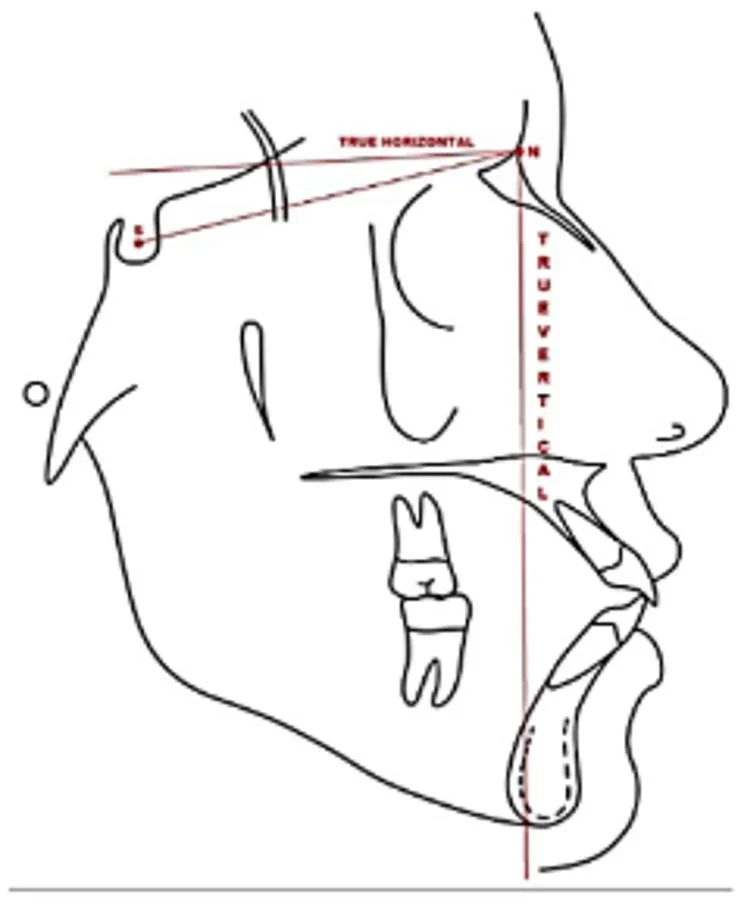
Schematic illustration of the true vertical plane (S: sella, N: nasion) [18]

The Jarabak Index was employed as an additional cephalometric parameter for evaluating vertical facial growth. This index represents the ratio of posterior facial height (Sella–Gonion) to anterior facial height (Nasion–Menton), derived from linear measurements obtained on the lateral cephalometric radiographs. According to the calculated percentage, individuals were classified into three categories: values below 62% indicated a hyperdivergent pattern, values between 62% and 65% represented a normodivergent pattern, and values above 65% corresponded to a hypodivergent pattern [18] (Fig. 5).

### FMA angle

**Fig. 6:**
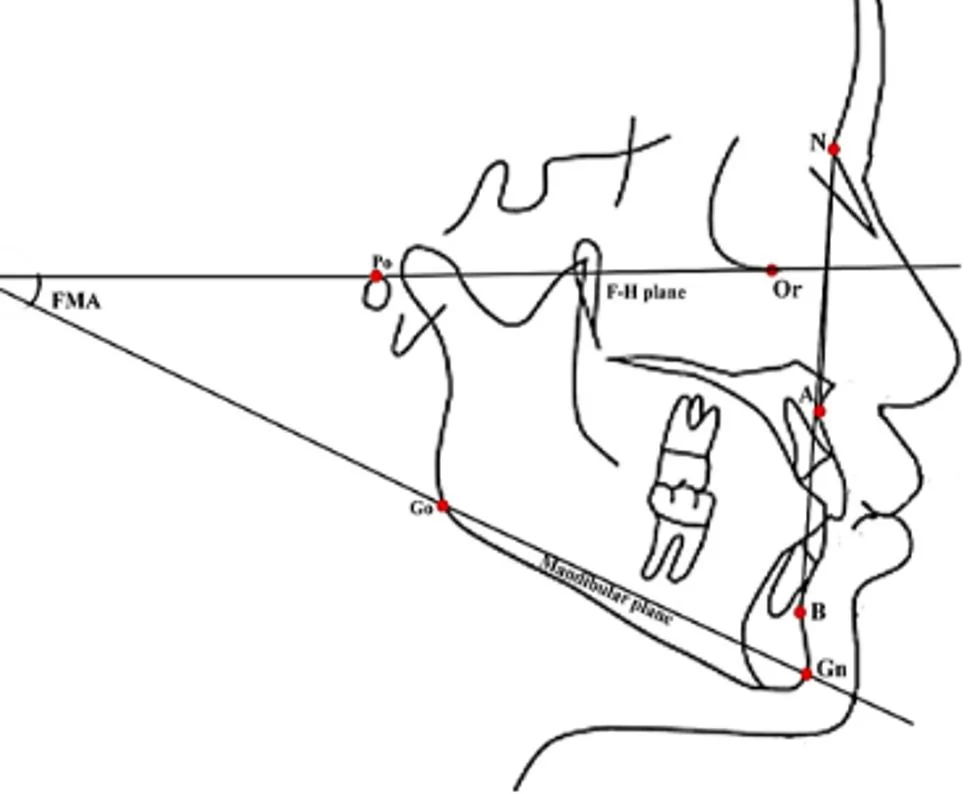
FMA angle between the Frankfort plane and the mandibular plane [19]

The FMA was used as a supplementary cephalometric parameter to characterize vertical facial growth. The angle was measured between the Frankfort horizontal plane (Porion (Po)–Orbitale (Or)) and the mandibular plane (Gonion (Go)–Gnathion (Gn)). Values within the range of 21° to 29° were considered normodivergent, whereas measurements exceeding 29° indicated a hyperdivergent pattern and those below 21° reflected a hypodivergent pattern [19]. (Fig. 6)

### Statistical analysis

Data were analyzed using Minitab version 21 and SPSS version 26. Descriptive statistics were reported as mean ± standard deviation (SD) or frequency and percentage. Spearman correlation analysis was used to assess the relationship between skeletal maturity and dental developmental stages within VFP groups. Ordinal logistic regression models were used to evaluate the associations of CA, skeletal maturity, gender, and VFP with U3, L7, and L8 developmental stages. Supplementary CVM × VFP interaction models were also performed. Measurement reliability was assessed using weighted kappa and intraclass correlation coefficient (ICC). A p-value < 0.05 was considered statistically significant.

## Results

### Measurement reliability

Radiographic assessments were performed by two orthodontic specialists. Measurement reliability indicated acceptable to good intra- and inter-observer agreement. For ordinal variables, the inter-observer and intra-observer weighted kappa values were 0.82 and 0.84 for CVM staging, 0.81 and 0.83 for U3 staging, 0.83 and 0.84 for L7 staging, 0.80 and 0.82 for L8 staging, and 0.81 and 0.83 for VFP classification, respectively. For cephalometric measurements used for VFP classification, the inter-observer and intra-observer ICC values were 0.79 and 0.81 for FMA, 0.75 and 0.78 for LAFH/TAFH, and 0.77 and 0.80 for Jarabak index, respectively. Overall, these findings indicated acceptable reproducibility of the radiographic staging and cephalometric measurements.

### Distribution of CA, gender, VFP, and developmental stages

The chronological age (CA) distribution of the participants demonstrated a moderate level of variability, with a mean CA of 12.95 ± 2.39 years. As shown in Tables 1 and 68.6% of participants were female, and 31.4% were male. Regarding vertical facial morphology, 55.3% of individuals exhibited a hyperdivergent pattern, while 22.1% and 22.6% were categorized as normodivergent and hypodivergent, respectively. CA generally increased across CVM stages and dental developmental stages, consistent with the expected developmental progression. In addition, CA was comparable across VFP groups (Kruskal-Wallis test, *p* = 0.358).

**Table 1:** CA distribution according to gender, VFP, CVM stage, and dental developmental stage

Variable	Group	*N* (%)	CA, Mean ± SD
Gender	Female	155 (68.6)	13.10 ± 2.44
	Male	71 (31.4)	12.62 ± 2.26
VFP	Hypodivergent	51 (22.6)	12.77 ± 2.51
	Normodivergent	50 (22.1)	12.80 ± 2.22
	Hyperdivergent	125 (55.3)	13.08 ± 2.42
CVM stage	CS1	26 (11.5)	10.15 ± 1.54
	CS2	21 (9.3)	10.52 ± 2.06
	CS3	31 (13.7)	11.68 ± 1.58
	CS4	45 (19.9)	12.56 ± 1.69
	CS5	53 (23.5)	14.30 ± 1.32
	CS6	50 (22.1)	15.12 ± 1.65
U3	I	4 (1.8)	08.25 ± 0.50
	II	16 (7.1)	09.13 ± 1.09
	III	22 (9.7)	10.18 ± 1.47
	IV	47 (20.8)	12.00 ± 1.38
	V	137 (60.6)	14.30 ± 1.61
L7	II	18 (08.0)	08.83 ± 1.10
	III	15 (06.6)	09.73 ± 0.80
	IV	51 (22.6)	11.63 ± 1.36
	V	77 (34.1)	13.49 ± 1.27
	VI	65 (28.8)	15.22 ± 1.45
L8	0	35 (15.5)	10.31 ± 2.34
	I	28 (12.4)	11.21 ± 1.97
	II	105 (46.5)	12.99 ± 1.49
	III	27 (12.0)	14.70 ± 1.03
	IV	23 (10.2)	15.48 ± 1.53
	V	7 (3.1)	16.86 ± 1.35
	VI	1 (0.4)	16.00

SD was not calculated for L8 stage VI due to *n* = 1

### Descriptive distribution of skeletal maturation and dental developmental stages

**Fig. 7:**
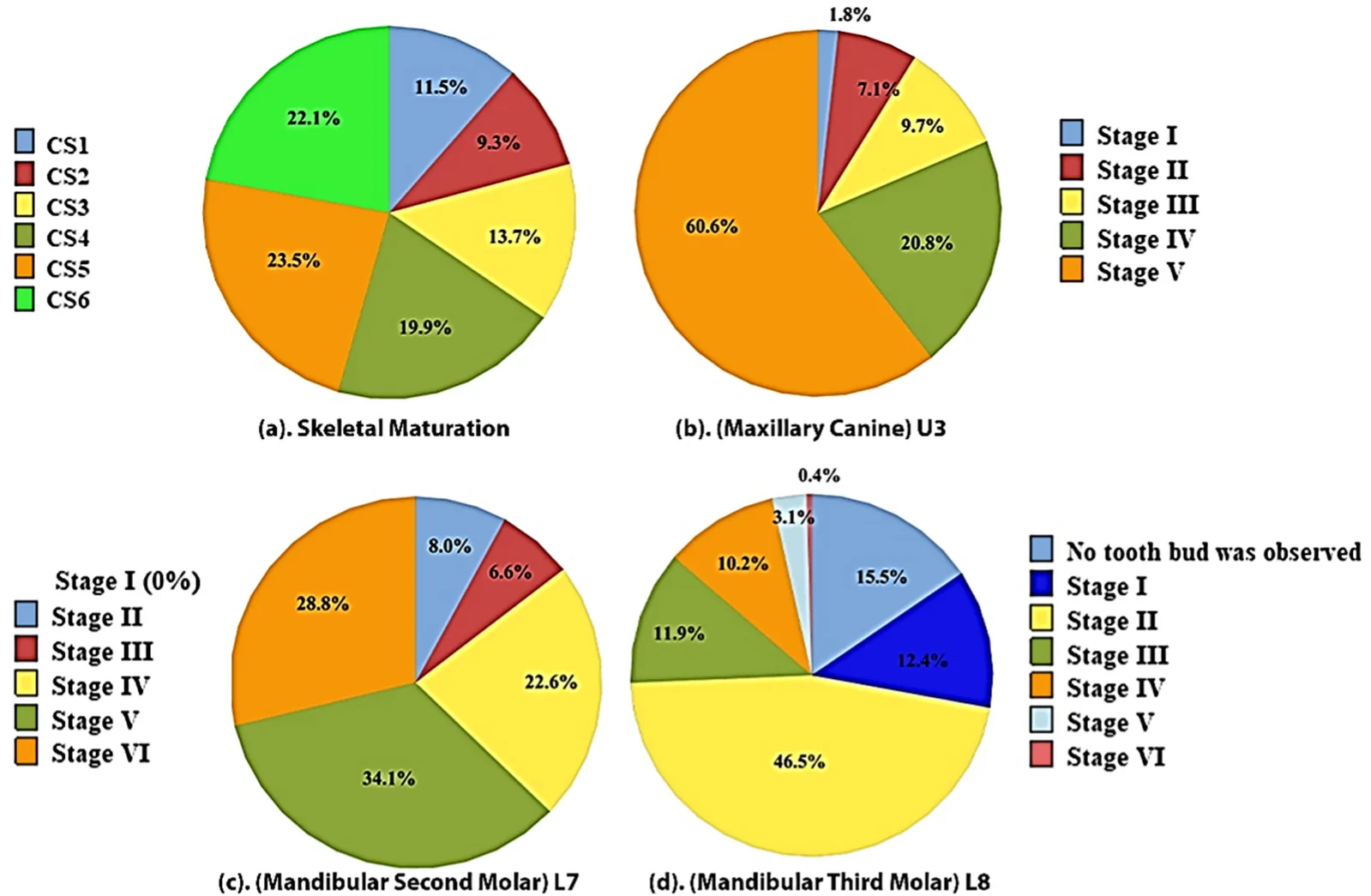
Proportions of participants across skeletal maturation stages and the developmental stages of U3, L7, and L8

The descriptive analyses provided a detailed overview of skeletal maturation and dental developmental stages. In this regard, the distribution of skeletal maturation showed that most participants were concentrated in the later CVM stages, with CS5 and CS6 together accounting for nearly half of the cohort, whereas CS2 represented the smallest proportion (Fig. 7a). Moreover, the three evaluated teeth exhibited distinct maturation patterns. In U3, Stage V (apex fully closed with a uniform periodontal ligament space) was the most prevalent stage (60.6%), whereas Stages I, II, and III accounted for 1.8%, 7.1%, and 9.7% of cases, respectively (Fig. 7b). L7 showed no cases in Stage I (0%; occlusal enamel complete and dentin formation begins). The highest proportions were observed in Stage V (34.1%; parallel canals with a partially open apex) and Stage VI (28.8%; fully closed apex with a uniform periodontal ligament space), indicating progression toward the later phases of root development (Fig. 7c). Finally, L8 exhibited the most immature developmental pattern among the three teeth. Stage II (crown completed to the cementoenamel junction) accounted for 46.5% of cases, and 15.5% showed no visible tooth bud. Only one individual (0.4%) was classified in Stage VI (fully closed apex with a uniform periodontal ligament space) (Fig. 7d).

### Correlation between skeletal maturity and dental developmental stages

Table 2 summarizes the Spearman correlation coefficients between skeletal maturity and the developmental stages of U3, L7, and L8 across the three VFPs. All correlations were positive and statistically significant (*p* < 0.001). Moderate to strong associations were observed for most measurements, particularly for L7, which demonstrated the highest correlation values within VFP subgroups. Moderate correlations were found primarily for L8. Overall, skeletal maturation showed a consistent positive relationship with dental developmental progression in all facial pattern subgroups.

**Table 2:** Relationship between skeletal maturation and dental developmental stages by VFP

Facial Pattern	Tooth	*N*	Spearman ρ	*p*-value
Hypodivergent	U3	51	0.663	< 0.001
	L7	51	0.769	< 0.001
	L8	51	0.558	< 0.001
Normodivergent	U3	50	0.614	< 0.001
	L7	50	0.705	< 0.001
	L8	50	0.627	< 0.001
Hyperdivergent	U3	125	0.643	< 0.001
	L7	125	0.684	< 0.001
	L8	125	0.575	< 0.001

U3: Maxillary Canine, L7: Mandibular 2nd Molar, L8: Mandibular 3rd Molar

To further evaluate whether VFP modified this association, supplementary fully adjusted ordinal logistic regression models including the CVM×VFP interaction term were performed (Table 3). Models were adjusted for CA, gender, skeletal maturity, VFP, and the CVM × VFP interaction term. Overall, the interaction analysis revealed that the CVM × Normodivergent term reached statistical significance for U3 and L7 (*p* = 0.047 and *p* = 0.049, respectively), whereas the CVM × Hyperdivergent term was not significant for U3 or L7 (*p* = 0.145 and *p* = 0.214, respectively). No significant CVM × VFP interaction was observed for L8.

**Table 3:** Interaction analysis of CVM and VFP in fully adjusted ordinal logistic regression models

Tooth	Interaction term	Estimate (β)	SE	Z	*p*-value
U3	CVM × Hyperdivergent	0.480	0.331	1.45	0.145
	CVM × Normodivergent	0.713	0.359	1.98	0.047
L7	CVM × Hyperdivergent	0.303	0.243	1.24	0.214
	CVM × Normodivergent	0.523	0.271	1.93	0.049
L8	CVM × Hyperdivergent	-0.181	0.210	-0.86	0.389
	CVM × Normodivergent	-0.026	0.238	-0.11	0.912

Hypodivergent VFP = Reference category

### Effects of skeletal maturity and CA on dental development

As shown in Table 4, CA showed a statistically significant association with dental developmental stage across all regression models and demonstrated the largest Wald statistics. For U3, CA was significantly associated with developmental stage (β = -1.240, Wald = 73.44, *p* < 0.001), whereas skeletal maturity showed a marginal but non-significant association (β = -0.266, *p* = 0.056). Neither the hyperdivergent pattern (β = 0.647, *p* = 0.122) nor the normodivergent pattern (β = 0.736, *p* = 0.130) differed significantly from the hypodivergent reference group.

**Table 4:** Effects of skeletal maturity, CA, and VFP on the development of U3, L7, and L8

Tooth		Predictor	Estimate (β)	SE	Wald	*p*-value	OR (95% CI)
U3	Skeletal Maturity		-0.266	0.139	3.64	0.056	0.77 (0.58–1.01)
	CA		-1.240	0.145	73.44	< 0.001	0.29 (0.22–0.38)
	Normodivergent		0.736	0.486	2.31	0.130	2.09 (0.81–5.41)
	Hyperdivergent		0.647	0.419	2.38	0.122	1.91 (0.84–4.35)
L7	Skeletal Maturity		-0.377	0.129	8.66	0.003	0.69 (0.53–0.88)
	CA		-1.236	0.128	91.63	< 0.001	0.29 (0.23–0.37)
	Normodivergent		0.298	0.424	0.49	0.482	1.35 (0.59–3.09)
	Hyperdivergent		0.400	0.359	1.24	0.265	1.49 (0.74–3.02)
L8	Skeletal Maturity		-0.111	0.121	0.85	0.356	0.89 (0.71–1.13)
	CA		-0.864	0.098	77.03	<0.001	0.42 (0.35–0.51)
	Normodivergent		-0.307	0.392	0.61	0.432	0.74 (0.34–1.58)
	Hyperdivergent		0.218	0.331	0.43	0.510	1.24 (0.65–2.38)

*SE* Standard Error, *CA* Chronological age, *OR* Odds Ratio, *CI* Confidence interval, *Hypodivergent* Reference group

In the L7 model, both CA and skeletal maturity were statistically significant. CA showed a significant association (β = -1.236, Wald = 91.63, *p* < 0.001), and skeletal maturity also contributed significantly (β = -0.377, Wald = 8.66, *p* = 0.003). In contrast, VFP showed no statistically significant independent association with L7 development (hyperdivergent: β = 0.400, *p* = 0.265; normodivergent: β = 0.298, *p* = 0.482).

For L8, CA remained the only statistically significant variable (β = -0.864, Wald = 77.03, *p* < 0.001). Skeletal maturity showed no significant association (β = -0.111, *p* = 0.356), and both hyperdivergent and normodivergent patterns showed no significant differences from the hypodivergent reference category (*p* > 0.05). Overall, these findings indicate that CA was consistently associated with dental developmental stage across U3, L7, and L8, whereas skeletal maturity remained significant only for L7, and VFP showed no statistically significant independent association in these models.

### Influence of skeletal maturity, gender, and VFP on U3, L7, and L8

As shown in Table 5, fully adjusted ordinal logistic regression models were used to assess the combined effects of CA, skeletal maturity, gender, and VFP on the developmental stages of U3, L7, and L8. Male and hypodivergent VFP were used as reference categories. For U3, the model showed a significant improvement over the null model (likelihood-ratio χ^2^ = 220.198, df = 5, *p* < 0.001). The goodness-of-fit statistics indicated acceptable model fit (Pearson χ^2^ = 275.451, df = 487, *p* = 1.000; Deviance χ^2^ = 198.381, df = 487, *p* = 1.000). CA was the only statistically significant predictor of U3 developmental stage (β = −1.276, *p* < 0.001). Skeletal maturity was not significantly associated with U3 development after adjustment for CA, gender, and VFP (β = −0.204, *p* = 0.168). Gender was also not significant (Female: β = −0.452, *p* = 0.224). Neither the hyperdivergent pattern (β = 0.556, *p* = 0.191) nor the normodivergent pattern (β = 0.724, *p* = 0.136) differed significantly from the hypodivergent reference group.

**Table 5:** Fully adjusted ordinal logistic regression analysis of chronological age, skeletal maturity, gender, and VFP on U3, L7, and L8 developmental stages

Tooth	Predictor	Estimate (β)	SE	Wald	*p*-value	OR (95% CI)
U3	Skeletal Maturity	−0.204	0.148	1.90	0.168	0.82 (0.61–1.09)
	CA	−1.276	0.149	73.27	< 0.001	0.28 (0.21–0.37)
	Gender (Female)	−0.452	0.372	1.49	0.224	0.64 (0.31–1.32)
	Normodivergent	0.724	0.486	2.22	0.136	2.06 (0.80–5.35)
	Hyperdivergent	0.556	0.425	1.72	0.191	1.74 (0.76–4.01)
L7	Skeletal Maturity	−0.390	0.136	8.24	0.004	0.68 (0.52–0.88)
	CA	−1.232	0.130	89.68	< 0.001	0.29 (0.23–0.38)
	Gender (Female)	0.097	0.325	0.09	0.765	1.10 (0.58–2.08)
	Normodivergent	0.309	0.426	0.53	0.467	1.36 (0.59–3.14)
	Hyperdivergent	0.422	0.365	1.32	0.249	1.52 (0.74–3.12)
L8	Skeletal Maturity	−0.136	0.127	1.14	0.285	0.87 (0.68–1.12)
	CA	−0.858	0.099	75.00	< 0.001	0.42 (0.35–0.51)
	Gender (Female)	0.198	0.305	0.42	0.515	1.22 (0.67–2.22)
	Normodivergent	−0.278	0.394	0.49	0.481	0.76 (0.35–1.64)
	Hyperdivergent	0.258	0.338	0.58	0.444	1.29 (0.67–2.51)

Gender (Male) and hypodivergent VFP were considered the reference groups

For L7, the fully adjusted model also showed a significant improvement over the null model (likelihood-ratio χ^2^ = 279.319, df = 5, *p* < 0.001). The goodness-of-fit statistics supported acceptable model fit (Pearson χ^2^ = 282.870, df = 487, *p* = 1.000; Deviance χ^2^ = 224.503, df = 487, *p* = 1.000). In this model, both CA (β = −1.232, *p* < 0.001) and skeletal maturity (β = −0.390, *p* = 0.004) were statistically significant predictors of L7 developmental stage. Gender was not significantly associated with L7 development (Female: β = 0.097, *p* = 0.765). VFP also showed no significant association, as neither the hyperdivergent pattern (β = 0.422, *p* = 0.249) nor the normodivergent pattern (β = 0.309, *p* = 0.467) differed significantly from the hypodivergent reference group.

For L8, the fully adjusted model showed a significant improvement over the null model (likelihood-ratio χ^2^ = 179.551, df = 5, *p* < 0.001). The goodness-of-fit results were Pearson χ^2^ = 1404.990, df = 733, *p* < 0.001 and Deviance χ^2^ = 335.520, df = 733, *p* = 1.000. CA was the only statistically significant predictor of L8 developmental stage (β = −0.858, *p* < 0.001). Skeletal maturity was not significant after adjustment for CA, gender, and VFP (β = −0.136, *p* = 0.285). Gender was not significantly associated with L8 development (Female: β = 0.198, *p* = 0.515). Similarly, neither the hyperdivergent pattern (β = 0.258, *p* = 0.444) nor the normodivergent pattern (β = −0.278, *p* = 0.481) showed a significant difference from the hypodivergent reference group. Overall, CA showed the most consistent association with dental developmental stage across the three teeth. After adjustment for CA, gender, and VFP, skeletal maturity remained independently associated only with L7 development, whereas gender and VFP showed no significant independent associations.

### Effect of VFP on dental maturation (Kruskal–Wallis test)

As shown in Table 6, Kruskal–Wallis tests were used to evaluate the effect of VFP on the developmental stages of U3, L7, and L8. For U3, the test statistic was H = 1.224 with 2 degrees of freedom (*p* = 0.542). For L7, the test resulted in H = 0.589 (df = 2, *p* = 0.745), and for L8, H = 0.690 (df = 2, *p* = 0.708). In all three teeth, the p-values exceeded the 0.05 threshold, indicating no statistically significant differences in developmental stage among the Hyperdivergent, Normodivergent, and Hypodivergent groups. Mean rank values were also comparable across facial patterns.

**Table 6:** Kruskal–Wallis test comparing dental developmental stages (U3, L7, L8) among VFP groups

Tooth	Facial pattern	*N*	Mean rank	H	df	*p*-value
U3	Hypodivergent	51	116.08	1.224	2	0.542
	Normodivergent	50	105.60			
	Hyperdivergent	125	115.61			
L7	Hypodivergent	51	111.38	0.589	2	0.745
	Normodivergent	50	108.73			
	Hyperdivergent	125	116.27			
L8	Hypodivergent	51	112.17	0.690	2	0.708
	Normodivergent	50	119.88			
	Hyperdivergent	125	111.49			

### Gender differences in dental and skeletal maturation

The potential influence of gender on dental and skeletal maturation was further explored using the Mann–Whitney U test, given the ordinal nature of the developmental stages. The test statistics are summarized in Table 7. For U3, a statistically significant difference was found between females and males (U = 4683.500, Z = − 2.050, *p* = 0.040). However, gender differences for L7 and L8 were not statistically significant (*p* = 0.087 and *p* = 0.249, respectively). Furthermore, a significant difference between females and males was observed for skeletal maturity (CVM) (U = 3673.500, Z = − 4.084, *p* < 0.001).

**Table 7:** Mann–Whitney U test comparing dental and skeletal maturation stages between females and males

Variable	Mann–Whitney U	Wilcoxon W	Z	*p*-value
U3	4683.500	7239.500	-2.050	0.040
L7	4751.000	7307.000	-1.713	0.087
L8	5005.500	7561.500	-1.154	0.249
Skeletal maturity	3673.500	6229.500	-4.084	< 0.001

## Discussion

This study revealed tooth-specific differences in the relationship between dental development and skeletal maturation. Although U3, L7, and L8 were positively correlated with CVM, skeletal maturity remained independently associated only with L7 after multivariable adjustment. VFP showed no significant independent main effect.

The results revealed that our sample predominantly consisted of females (68.6%) and individuals with a hyperdivergent pattern (55.3%). While skewed gender distribution is common in orthodontic research, it may reflect referral patterns rather than population prevalence [20]. Observations by Harris et al. indicated that girls are more likely to seek orthodontic treatment for esthetic issues, resulting in a higher uptake of orthodontic services among females compared to males [21]. Similarly, Shayan et al. reported a higher proportion of females in their Iranian sample, which aligns with our study. However, in their study, the normodivergent pattern was more prevalent (47.2%), with only 41.4% of participants exhibiting a hyperdivergent pattern [22]. This discrepancy is most likely attributable to differences in the study populations, measurement methodologies, and the age–gender composition of the samples.

Nearly half of the participants were classified in the late CVM stages (CS5–CS6), corresponding to late/post-peak skeletal maturation stages. These findings are consistent with the results of Felemban [23], Kumar et al. [7], and Trakiniene et al. [5], who similarly reported a predominance of late skeletal maturation stages among adolescents in their samples. Additionally, U3 was predominantly in Stage V (60.6%), reflecting advanced dental maturation and a close synchrony with the terminal phases of skeletal growth. Kumar et al. [7] likewise demonstrated that the permanent maxillary canine most frequently reached its highest calcification stage (DI Stage H) at CS5–CS6, reinforcing that Stage V of the maxillary canine typically corresponds to late skeletal maturation. Moreover, L7 was most frequently observed in Stage V (34.1%), indicating that mandibular second molar development was mainly concentrated in the later phases of root formation. Consistently, Trakinienė et al. reported that the mandibular second molar most often remained in intermediate developmental stages during early and mid-skeletal maturation, reaching full root formation only in the later skeletal phases (CS4–CS6) [5]. Likewise, Trakinienė et al. also reported that the mandibular third molar typically remained in early or intermediate developmental stages (LM3-1 to LM3-4) across CS1–CS4 and reached full root formation (LM3-6) only in a small subset of individuals at the later skeletal stages (CS5–CS6) [5]. This pattern corroborates our finding that L8 (46.5% in Stage II; 0.4% in Stage VI) showed a less advanced developmental pattern relative to the other evaluated teeth.

The findings also demonstrated moderate to strong positive correlations between skeletal maturity and dental developmental stages, with L7 showing the highest correlation values, followed by U3, whereas L8 exhibited weaker but still significant correlations. Unadjusted gender comparisons showed a significant difference for U3 and skeletal maturity; however, gender was not independently associated with dental developmental stages in the fully adjusted models. These findings are consistent with previous reports by Firouzinia et al. [12], Al-Balbeesi et al. [8], and Jourieh et al. [24]. In this regard, Al-Balbeesi et al. reported a positive association between CVM and the developmental stages of the canine and third molar in a Saudi population, although the correlation coefficients were comparatively lower (0.166–0.612). These differences may reflect racial and methodological variations between studies. Nonetheless, despite not evaluating VFPs separately, their findings align with the present study in confirming that skeletal maturation progresses in parallel with maxillary canine development [8]. The observed correlations of U3 and L7 with skeletal maturity suggest that their developmental progression coincides with pubertal skeletal maturation, a pattern that has been widely reported in both regional [12] and international cohorts [8]. Additionally, the association between skeletal maturation and canine development may partly reflect shared biological processes occurring during puberty. IGF-1 has been shown to correlate with skeletal maturity and residual mandibular growth [25]. In contrast, the weaker correlation for L8 reflects the inherent variability and later developmental timing of the mandibular third molar, making it a less reliable indicator of skeletal maturity, as recent studies have emphasized its diagnostic limitations [26, 27]. The limited unadjusted gender difference observed for U3 is also consistent with systematic reviews by Khan et al., indicating that gender-related differences in dental calcification are typically modest and tooth-specific, often diminishing in posterior segments [28].

Building on the preceding findings, VFP showed no independent significant association with dental developmental stages as a main effect in the fully adjusted models. This suggests that, although vertical craniofacial morphology remains clinically relevant in orthodontic growth assessment, its contribution to dental maturation appears limited when CA, skeletal maturity, and gender are considered simultaneously. This interpretation is broadly consistent with previous studies reporting that dental development is more closely related to systemic maturational factors than to vertical facial morphology [29–31]. However, Gottimukkala et al. argued that CA alone is not sufficiently dependable and should be interpreted alongside facial growth patterns to improve the accuracy of estimating the pubertal growth spurt [32]. Because some previous studies have focused on relatively narrow age ranges (7.5–12 years), they may have been less able to capture the full variability associated with pubertal development [32], underscoring the need for further research on the influence of age on the relationship between skeletal maturation and dental calcification. In the present study, CA showed the most consistent association with dental developmental stage, whereas skeletal maturity retained an independent association only for L7. The prominent role of L7 is in line with Litsas and Lucchese [33] and Cossellu et al. [34], who reported significant correlations between dental calcification and CVM stages and found that second molars showed the highest correlation with skeletal maturation. These findings support a combined maturational assessment rather than reliance on a single indicator.

The supplementary interaction analysis indicated that the skeletal–dental maturation relationship may vary across selected VFP subgroups, particularly for U3 and L7. This finding is relevant because VFP did not show a consistent independent main effect in the fully adjusted models, suggesting that vertical facial morphology may not directly determine dental maturation but may influence how skeletal and dental maturation correspond in some subgroups. This interpretation is broadly consistent with previous studies reporting that dental development is more closely related to systemic maturational factors than to vertical facial morphology [13, 30].

For U3, the significant positive correlations with skeletal maturity observed in the present study support a parallel developmental pattern between maxillary canine calcification and skeletal maturation, consistent with previous studies reporting significant associations between maxillary canine developmental stages and CVM stages [5, 7, 12]. However, after adjustment for CA, gender, and VFP, skeletal maturity was no longer independently associated with U3 development. This attenuation may reflect the substantial overlap between CA and CVM as interrelated markers of biological maturation, consistent with previously reported correlations among CA, dental maturation, and cervical vertebral maturation [13]. Accordingly, U3 may serve as a useful complementary marker of maturation; however, its association with skeletal maturity did not remain independent of CA in the present cohort.

Notably, L7 was the only evaluated tooth for which skeletal maturity remained independently associated with developmental stage after adjustment for CA, gender, and VFP. This finding may partly reflect the closer temporal correspondence between mandibular second molar root development and the pubertal growth period. Previous studies have shown that intermediate L7 calcification stages coincide with successive phases of cervical vertebral maturation and that mandibular second molars exhibit particularly strong correlations with CVM stages [5, 31, 32]. In the present study, the broader distribution of L7 across intermediate and advanced developmental stages may also have provided greater discriminatory capacity than U3, for which most participants had already reached the final stage, or L8, which showed later and more variable development. Accordingly, L7 calcification may provide useful complementary information for maturational assessment when interpreted alongside CA and CVM, rather than being used as a stand-alone indicator.

For L8, the positive correlations observed within the VFP-stratified analyses did not persist after multivariable adjustment, with CA emerging as the only independently associated variable. This finding should not be interpreted as evidence of a complete dissociation between mandibular third molar development and skeletal maturation; rather, it suggests that L8 provided limited maturational information beyond that already captured by CA in the present cohort. Several factors may account for this attenuation. First, mandibular third molar development extends over a considerably broader developmental interval than the circumpubertal window represented by CVM, beginning in late childhood and frequently continuing into early adulthood [35]. Consequently, individuals within the same L8 developmental stage may differ substantially in skeletal maturity, thereby weakening its ability to discriminate between successive CVM stages. Second, the relationship between third molar mineralization and CVM appears to vary across demographic subgroups; previous work has reported a strong association in males but a substantially weaker association in females, suggesting that L8–CVM correspondence may not be equally stable across populations or sexes [36].

Third, the marked concentration of participants in the earlier L8 stages in the present sample, together with the very small number reaching complete apical closure, may have restricted stage variability and reduced the statistical capacity to detect an independent association after adjustment. In addition, the goodness-of-fit statistics for the L8 ordinal regression model produced divergent results. Although the deviance test did not indicate a lack of fit, the significant Pearson statistic suggested some disagreement between the observed and model-predicted frequencies. This discrepancy may be attributable to the sparse and markedly imbalanced distribution of the L8 outcome categories, particularly the presence of only one participant in Stage VI, because the Pearson statistic can be sensitive to small expected cell frequencies.

Finally, because CA, dental maturation, and CVM represent interrelated dimensions of biological development, the inclusion of CA in the model may have absorbed much of the shared maturational information underlying the unadjusted L8–CVM association [13].This interpretation is consistent with previous evidence showing that the correspondence of mandibular third molar calcification with CVM is weaker than that observed for U3 and particularly L7 [5]. Thus, although L8 may remain informative for the assessment of later adolescent development, the present findings do not support its use as a stand-alone indicator of pubertal skeletal maturation.

### Limitations of the Study

This study has some limitations. First, the sample was obtained from a single university-based orthodontic clinic, which may limit the generalizability of the findings to the general adolescent population. Second, the sample distribution was imbalanced, with a higher proportion of female participants and hyperdivergent facial patterns. Third, because of the cross-sectional design, growth changes over time could not be directly evaluated. Finally, although measurement reliability was assessed, manual radiographic staging and cephalometric measurements remain partly examiner-dependent. Future multicenter longitudinal studies with more balanced samples are recommended.

## Conclusion

This study demonstrated a clear association between dental developmental stages and skeletal maturation in Iranian children and adolescents, supporting the complementary value of these indicators in growth assessment. In the fully adjusted models, CA appeared to be a key determinant of dental developmental stage across U3, L7, and L8, while skeletal maturity retained an independent association only with L7 development. VFP showed no independent significant association with dental maturation after adjustment for CA, skeletal maturity, and gender, suggesting that vertical craniofacial morphology may have a limited role in dental developmental status when major maturational factors are considered. Among the evaluated teeth, L7 showed the most consistent correspondence with skeletal maturation and may therefore serve as a useful adjunctive indicator for maturational assessment. Overall, these findings support an integrated approach combining CA, CVM stage, and selected dental developmental indicators, which may contribute to individualized growth evaluation and orthodontic treatment timing.

## Data Availability

The datasets generated and/or analyzed during the current study are available from the corresponding author on reasonable request.

## Abbreviations

U3: Maxillary canine
L7: Mandibular second molar
L8: Mandibular third molar
VFP: Vertical facial pattern
VFPs: Vertical facial patterns
LAFH: Lower anterior facial height
TAFH: Total anterior facial height
FMA: Frankfort–mandibular plane angle
CVM: Cervical vertebral maturation
CVMS: Cervical vertebral maturation staging
CA: Chronological age
CS: Cervical stage
PLUM: Polytomous universal model
SD: Standard deviation
SE: Standard error
OR: Odds ratio
df: Degrees of freedom
IGF-1: Insulin-like growth factor-1
